# Unveiling Psychiatric Manifestations in a Case of Moyamoya Disease

**DOI:** 10.7759/cureus.89515

**Published:** 2025-08-06

**Authors:** Shahad N Alameeri, Meera A Alshehhi, Hussain Atraqchi, Mohsen M Elshamy

**Affiliations:** 1 Psychiatry, Al Amal Psychiatric Hospital, Emirates Health Services, Dubai, ARE; 2 Emergency Department, Rashid Hospital, Dubai Health, Dubai, ARE; 3 Psychiatry, Al Jalila Children's Specialty Hospital, Dubai Health, Dubai, ARE; 4 Psychiatry, Rashid Hospital, Dubai Health, Dubai, ARE

**Keywords:** digital subtraction angiography, moyamoya disease, psychosis, stroke, vasculitis

## Abstract

Moyamoya disease (MMD) is a rare and progressive cerebrovascular disorder characterized by stenosis of the internal carotid arteries and their major branches, leading to the development of abnormal collateral vessels. While MMD is traditionally associated with ischemic and hemorrhagic strokes, there is increasing recognition of the psychiatric symptoms that can accompany the disease, which significantly impact patient outcomes and complicate management. This case report presents a 30-year-old female with a history of recurrent ischemic strokes, hypertension, diabetes, and dyslipidemia, who initially presented with neurological symptoms including headache, left-sided weakness, and facial deviation. Along with these neurological signs, the patient developed psychiatric symptoms, including depression and later, psychosis. After referral to the psychiatry liaison team, the patient was diagnosed with post-stroke depression, followed by the development of persecutory delusions and grandiosity. Psychiatric treatment with selective serotonin reuptake inhibitors (SSRIs) and antipsychotics led to significant improvement in mood and resolution of psychotic symptoms. This case highlights the importance of early identification and management of psychiatric manifestations in MMD patients. A multidisciplinary approach, incorporating both neurological and psychiatric interventions, is essential to optimizing patient care and improving outcomes.

## Introduction

Moyamoya disease (MMD) is a rare and progressive cerebrovascular disorder characterized by stenosis of the internal carotid arteries and proximal anterior and middle cerebral arteries, resulting in the formation of abnormal network of collateral vessels [1,2]. Moyamoya means “puff of smoke” in Japanese and is used to describe the tangled appearance of tiny vessels compensating for the reduced blood flow in the major vessels of the anterior circulation of the brain [3-5]. This disease often leads to severe neurological complications, including ischemic and hemorrhagic strokes [1,6,7]. While the focus has traditionally been on these neurological manifestations, emerging evidence suggests that MMD can also present with significant psychiatric symptoms [8,9]. Psychiatric manifestations in MMD, though less commonly reported, can include mood disorders, psychosis, and cognitive impairments [6,10]. These symptoms may arise due to ischemic damage affecting brain regions involved in mood regulation and cognition [11]. Diagnosis of MMD relies on advanced imaging techniques, with digital subtraction angiography (DSA) being the gold standard [1]. Early intervention, including revascularization surgery, is crucial for managing symptoms and preventing further cerebrovascular events [8,12,13].

## Case presentation

**Figure 1:**
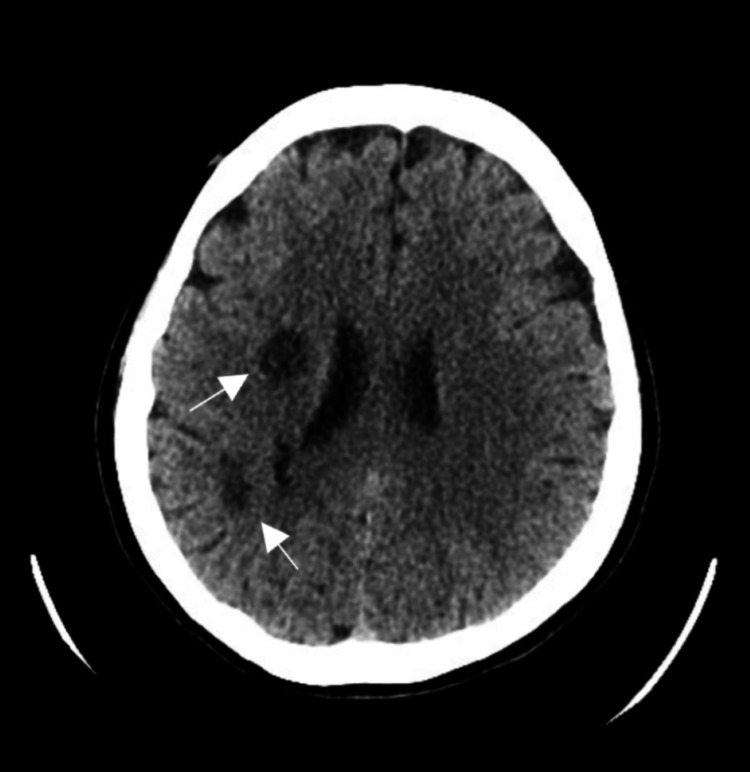
Plain CT brain showed multiple well-defined hypodensities in the right fronto-temporo-parietal regions, suggestive of subacute or chronic infarcts.

A 30-year-old female from the Philippines, with a history of recurrent ischemic strokes, hypertension, diabetes mellitus, and dyslipidemia, presented to the Emergency Department with a headache, left-sided weakness, and facial deviation that began two days prior to her presentation. Neurological examination revealed left-sided facial drooping, decreased sensation and strength in the upper and lower limbs, and slurred speech. The patient had experienced intermittent numbness and weakness over the past month. Routine laboratory tests were normal, and a plain CT brain showed multiple well-defined hypodensities in the right fronto-temporo-parietal regions, suggestive of subacute or chronic infarcts (Figure 1).

**Figure 2:**
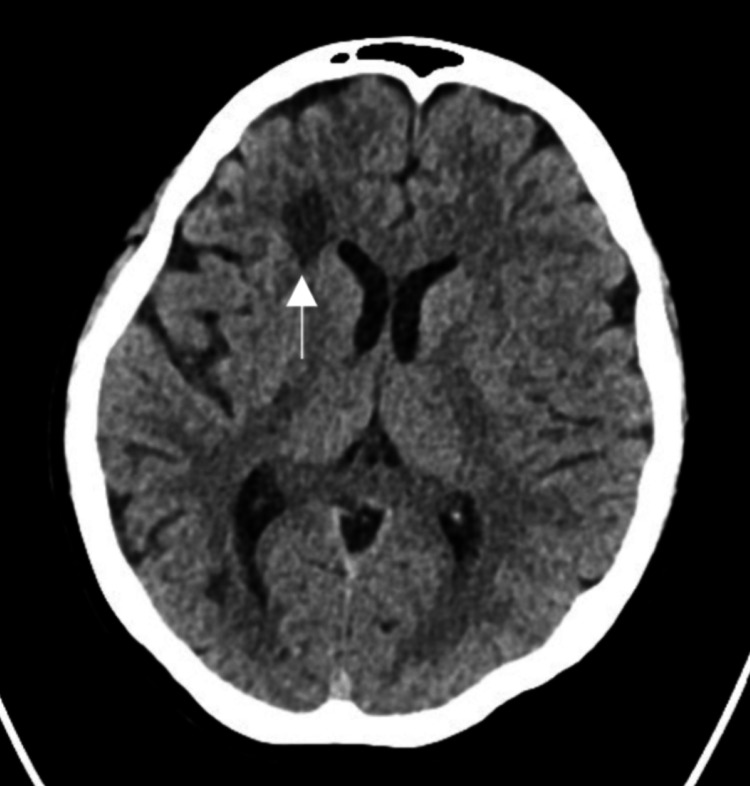
An urgent CT brain showed a new hypodensity in the right frontal lobe.

A CT Brain Angiogram indicated inflammatory vasculitis with narrowing in the supraclinoid portion of the right internal carotid artery and the M1 segment of the right middle cerebral artery, where the plan was to administer methylprednisolone 1 g IV for five days, then switch to oral prednisolone 60 mg and gradually taper the dosage. The medical team observed that the patient was tearful and anxious, leading to a referral to the psychiatry liaison team. Initial psychiatric assessment revealed low mood, helplessness, hopelessness, decreased appetite, insomnia, and decreased concentration over the past three days, without psychotic features. The patient was diagnosed with post-stroke depression and started on sertraline 25 mg daily and lorazepam 1 mg as needed for insomnia. Three days later, the patient showed significant improvement in mood, sleep, and appetite. She was more hopeful and motivated to return to daily activities, and no psychotic features were observed. The diagnosis was updated to adjustment disorder with depressed mood, and sertraline was increased to 50 mg daily. The patient continued to sleep well and no longer required lorazepam. On the eighth day of admission, the patient developed severe dizziness and blurring of vision. An urgent CT brain showed a new hypodensity in the right frontal lobe (Figure 2).

**Figure 3:**
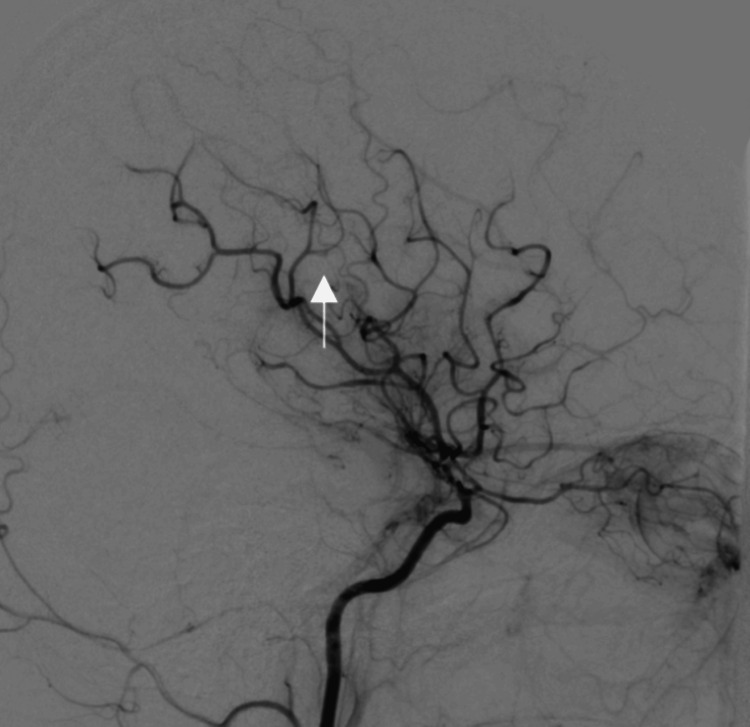
Angiography (DSA) revealed severe irregular narrowing of the right supraglenoid internal carotid artery (ICA), the M1 segment of the right middle cerebral artery (MCA), and the A1 segment of the right anterior cerebral artery (ACA), with numerous basal collaterals. The left ICA appeared normal, and no aneurysm or vascular malformation was detected.

On the 20th day, the psychiatry liaison team was consulted due to the patient exhibiting bizarre thoughts. She reported persecutory delusions and grandiosity, believing she had a special connection with God and that a neighboring patient was attempting to strangle her. Her mood was irritable, and she refused to follow hospital routines. The mental status examination revealed appropriate eye contact, orientation, and coherent thought form but persecutory delusions and grandiosity. Quetiapine 25 mg twice daily was initiated, and sertraline was discontinued. The patient was monitored for progression or resolution of psychotic symptoms. Over the next few days, the patient's delusions resolved, her mood stabilized, and she showed no signs of mania or agitation. She adhered to her medication regimen and expressed optimism about her health. The patient was discharged from the Psychiatry Department with a plan for follow-up care. A multidisciplinary meeting involving radiology, neurology, and internal medicine teams was held. DSA revealed severe irregular narrowing of the right supraglenoid internal carotid artery (ICA), the M1 segment of the right middle cerebral artery (MCA), and the A1 segment of the right anterior cerebral artery (ACA), with numerous basal collaterals. The left ICA appeared normal, and no aneurysm or vascular malformation was detected (Figure 3).

The neurology team confirmed the diagnosis of isolated moyamoya disease and planned to discontinue steroid therapy, initiate dual antiplatelet therapy for four weeks, and then switch to single antiplatelet therapy. The patient was referred to the neurosurgery team for possible recanalization.

## Discussion

MMD is a rare cerebrovascular disorder that primarily manifests with ischemic and hemorrhagic strokes due to progressive stenosis of the internal carotid arteries and their major branches [1,2,4,6]. Although traditionally focused on its neurological consequences, such as ischemic events, recent studies underscore the significance of psychiatric symptoms in MMD patients. Psychiatric disturbances, including mood disorders, cognitive impairments, and psychosis, are increasingly recognized as part of the disease's multifaceted presentation, often complicating diagnosis and management [8].

This case illustrates how psychiatric symptoms can emerge or worsen after neurological events in MMD, highlighting the need for integrated care. In our patient, depression appeared initially, followed by psychosis, including persecutory delusions and grandiosity, after a series of ischemic events. This progression is consistent with findings from other studies, where psychiatric symptoms in MMD often emerge following cerebrovascular incidents, supporting the hypothesis that ischemic damage to regions responsible for emotional regulation may contribute to these symptoms. It is increasingly understood that psychiatric disturbances in MMD are not merely a psychological reaction to the disease but are likely influenced by direct ischemic damage to critical brain areas, including the frontal and temporal lobes, which are involved in mood and cognitive functions [8]. The literature has shown that mood disorders, especially depression, are the most common psychiatric symptoms in MMD, with psychosis appearing less frequently but still significant. These psychiatric manifestations can significantly impact patients' quality of life and complicate the management of the disease, as untreated psychiatric symptoms may interfere with adherence to medical regimens and rehabilitation efforts. In this case, psychiatric intervention with selective serotonin reuptake inhibitors (SSRIs) and antipsychotics led to a notable improvement in the patient's mood and resolution of psychotic symptoms, which is consistent with existing treatment protocols for mood disorders in MMD patients [6]. Recent studies on MMD emphasize that psychiatric symptoms can appear either before or after ischemic events, with some cases of depression or cognitive impairment presenting even before the onset of strokes. This suggests that psychiatric symptoms in MMD may be part of a broader neurovascular process, where the brain’s vulnerability to ischemia may manifest as both neurological and psychiatric disturbances.

Furthermore, research has shown that early recognition and treatment of psychiatric symptoms in MMD are essential for improving both neurological recovery and overall patient well-being [1]. In this case, the prompt psychiatric assessment and treatment led to significant recovery, highlighting the effectiveness of an integrated, multidisciplinary approach. The psychological impact of living with a chronic, progressive condition like MMD cannot be overstated. While ischemic events contribute to the development of psychiatric symptoms, the ongoing stress of managing such a debilitating illness likely exacerbates these effects. Recent studies have shown that untreated psychiatric symptoms in MMD, particularly depression and psychosis, can significantly worsen overall outcomes. Without intervention, these symptoms can lead to cognitive decline, decreased functional capacity, and reduced adherence to treatment protocols, which may further impact neurological recovery [6]. In our case, the prompt initiation of psychiatric treatment led to significant mood stabilization and resolution of psychotic symptoms, supporting the importance of early psychiatric intervention in improving both psychological and neurological outcomes.

## Conclusions

In this case report we present a 30-year-old female with history of recurrent strokes, presenting to the emergency department with new neurological deficits and neuropsychiatric symptoms. Initial imaging and angiography suggested vasculitis, leading to steroid treatment. However, further assessment with DSA confirmed a diagnosis of moyamoya disease, which showed a progressive narrowing of cerebral arteries, prompting a change to antiplatelet therapy and a neurosurgical referral. During admission, the patient experienced symptoms of post-stroke depression, an adjustment disorder, and transient psychosis, all managed with antidepressants, antipsychotics and benzodiazepines. This case underscores the importance of thorough vascular evaluation in stroke patients with neuropsychiatric symptoms and highlights moyamoya disease as a rare but critical diagnosis requiring neurosurgical and neuromedical management.
